# Expression of genes involved in hepatic carnitine synthesis and uptake in dairy cows in the transition period and at different stages of lactation

**DOI:** 10.1186/1746-6148-8-28

**Published:** 2012-03-14

**Authors:** Gloria Schlegel, Janine Keller, Frank Hirche, Stefanie Geißler, Frieder J Schwarz, Robert Ringseis, Gabriele I Stangl, Klaus Eder

**Affiliations:** 1 Institute of Animal Nutrition and Nutrition Physiology, Justus-Liebig-Universität Giessen, Heinrich-Buff-Ring 26-32, D-35392 Giessen, Germany; 2 Institute of Agricultural and Nutritional Sciences, Martin-Luther-Universität Halle, Von-Danckelmann-Platz 2, D-06120 Halle, Saale, Germany; 3 Animal Nutrition, Technische Universität München, Liesel-Beckmann-Strasse 6, D-85354 Freising, Germany

## Abstract

**Background:**

In rodents and pigs, it has shown that carnitine synthesis and uptake of carnitine into cells are regulated by peroxisome proliferator-activated receptor α (PPARA), a transcription factor which is physiologically activated during fasting or energy deprivation. Dairy cows are typically in a negative energy balance during early lactation. We investigated the hypothesis that genes of carnitine synthesis and uptake in dairy cows are enhanced during early lactation.

**Results:**

mRNA abundances of PPARA and some of its classical target genes and genes involved in carnitine biosynthesis [trimethyllysine dioxygenase (TMLHE), 4-N-trimethylaminobutyraldehyde dehydrogenase (ALDH9A1), γ-butyrobetaine dioxygenase (BBOX1)] and uptake of carnitine [novel organic cation transporter 2 (SLC22A5)] as well as carnitine concentrations in liver biopsy samples of 20 dairy cows in late pregnancy (3 wk prepartum) and early lactation (1 wk, 5 wk, 14 wk postpartum) were determined. From 3 wk prepartum to 1 wk postpartum, mRNA abundances of PPARΑ and several PPARΑ target genes involved in fatty acid uptake, fatty acid oxidation and ketogenesis in the liver were strongly increased. Simultaneously, mRNA abundances of enzymes of carnitine synthesis (TMLHE: 10-fold; ALDH9A1: 6-fold; BBOX1: 1.8-fold) and carnitine uptake (SLC22A5: 13-fold) and the concentration of carnitine in the liver were increased from 3 wk prepartum to 1 wk postpartum (*P *< 0.05). From 1 wk to 5 and 14 wk postpartum, mRNA abundances of these genes and hepatic carnitine concentrations were declining (*P *< 0.05). There were moreover positive correlations between plasma concentrations of non-esterified fatty acids (NEFA) and hepatic carnitine concentrations at 1 wk, 5 wk and 14 wk postpartum (*P *< 0.05).

**Conclusions:**

The results of this study show for the first time that the expression of hepatic genes of carnitine synthesis and cellular uptake of carnitine is enhanced in dairy cows during early lactation. These changes might provide an explanation for increased hepatic carnitine concentrations observed in 1 wk postpartum and might be regarded as a physiologic means to provide liver cells with sufficient carnitine required for transport of excessive amounts of NEFA during a negative energy balance.

## Background

Carnitine (3-hydroxy-4-*N, N, N*-trimethylaminobutyric acid) is an essential metabolite that has a number of indispensable functions in intermediary metabolism. The most important function lies in its role in the transport of activated long-chain fatty acids (acyl groups) from the cytosol into the mitochondrial matrix where β-oxidation takes place [1]. Carnitine is derived from dietary sources and synthesized endogenously from trimethyllysine (TML), which is released upon protein degradation. The released TML is further oxidized to γ-butyrobetaine (BB) by the action of trimethyllysine dioxygenase (TMLHE), 3-hydroxy-N-TML aldolase and 4-N-trimethylaminobutyraldehyde dehydrogenase (ALDH9A1). In the final biosynthetic step, BB is hydroxylated by γ-butyrobetaine dioxygenase (BBOX1) to form carnitine. In cattle this last step occurs only in liver and kidney [2]. Tissues which are not capable of producing carnitine depend on the uptake of carnitine from blood by novel organic cation transporters (OCTN), particularly novel organic cation transporter 2 (SLC22A5) which is the physiologically most important carnitine transporter [3,4]. Studies in rodents and pigs demonstrated that carnitine biosynthesis and uptake of carnitine from blood into cells by SLC22A5 are directly regulated by peroxisome proliferator-activated receptor α (PPARA), a transcription factor which plays a central role in the adaptation of metabolism to energy deficiency [5]. In these species, activation of PPARA such as induced by fasting or treatment with synthetic agonists led to increased tissue carnitine concentrations due to an increased rate of biosynthesis and an increased uptake of carnitine from blood into tissues [6-10].

In dairy cows, the transition from late pregnancy to early lactation is associated with severe metabolic adaptations. Production of milk leads to a strong increase of the energy requirement, which however cannot be met as the food intake capacity is limited. Thus, during early lactation, dairy cows are typically in a negative energy balance which is compensated by the mobilization of non-esterified fatty acids (NEFA) from adipose tissue. NEFA are transported by binding with serum albumin and are taken up by fatty acid transporters into tissues, mainly the liver [11]. Studies in rodents have clearly established that NEFA taken up into the liver are able to bind to and activate PPARA [12,13]. In contrast to the large body of literature in non-ruminants, very little work has been conducted to define the specific effects or mechanisms of PPARA in cattle liver so far. However, a recent study using clofibrate as a synthetic agonist in weaned calves showed that PPARA is functional in cattle liver [14]. Moreover, it has been shown that long chain fatty acids are able to activate PPARA also in bovine cells [15]. In accordance with this finding, a negative energy balance in dairy cattle, either occurring physiologically during early lactation or induced by feed restriction, was associated with an up-regulation of several PPARA target genes involved in fatty acid oxidation or ketosis in the liver, indicative of an activation of PPARA [16,17].

To our knowledge, the regulation of carnitine homeostasis in dairy cattle has been less investigated. However, it has been found that hepatic carnitine concentration in dairy cows is increasing during the transition from late pregnancy to early lactation [18,19]. That finding and the assumption that genes involved in carnitine homeostasis in dairy cows might be regulated by PPARA such as in other species, prompted us to the hypothesis that hepatic genes of carnitine synthesis and uptake of carnitine are up-regulated during early lactation in dairy cows. To investigate this hypothesis, we determined mRNA abundances of the relevant genes involved in carnitine synthesis as well as carnitine concentrations in liver biopsy samples of dairy cows in late pregnancy and early lactation.

## Methods

The animal experiment was conducted at the Agricultural Experimental Station Hirschau of the Technical University of Munich, Germany. It was approved by the Bavarian state animal care and use committee.

### Animals and feeding

This study included twenty Holstein cows (four primi- and sixteen multiparous, 2.7 ± 0.3 parities, mean ± SE) as experimental animals with an experimental period from 3 wk prepartum until 14 wk postpartum. The animals were housed in a playpen. Feeding was composed of a partial mixed ration (PMR) for ad libitum intake of basic feed and separately allocated concentrates [supplemental concentrate (SUPP), 0.63 kg DM/d for each cow; individual concentrate (CONC), individual access]. PMR consisted (dry matter, DM, basis) of 33.7% grass silage, 44.9% maize silage, 6.4% hay and 14.9% concentrate while SUPP contained (DM basis) 24.4% soybean meal, 48.3% grain maize and 27.3% rumen-protected fat supplement. With an assumed dry matter intake of 16 kg of PMR/d and the alloted amount of SUPP, the calculated nutrient supply covered the energy and protein requirements for 23 kg of milk/d. CONC was individually allocated at four computer-operated feeding stations with an automatic feeding program (DeLaval Alpro, Glinde, Germany). CONC was composed of 24.8% grain maize, 21.8% wheat, 20.1% soybean meal, 15.2% dried sugar beet pulp with molasses, 14.9% barley and 3.2% vitamin-mineral premix including limestone (DM basis). The allocation of CONC was increased from 1.2 to 8.0 kg of DM/d during the first 42 d of lactation, and thereafter, it was dependent on the milk performance of the individual cow. Daily intakes of PMR and CONC were recorded for each individual cow. The cows were generally in a good health condition, although four cows had slight metabolic diseases (subclinical ketosis, subclinical acidosis) and nine cows suffered temporarily from mastitis. All used feed components were sampled and analyzed for DM content, for crude nutrients, crude ash, crude fibre and crude fat according to [20], and crude protein by Dumas method. According to the German Society of Nutrition Physiology [21], the net energy content (MJ NEL) and the available CP at the duodenum were calculated. Nutrient concentrations and energy content of all feed components are shown in Table 1.

**Table 1:** Nutrient values of experimental feedstuff

	PMR	CONC	SUPP
Energy* (MJ NE_L_/kg of DM)	6.45	8.00	12.8

Crude fibre (g/kg of DM)	214	67	69

Crude ash (g/kg of DM)	81	72	49

Crude fat (g/kg of DM)	32	20	303

CP (g/kg of DM)	129	184	140

Available CP (g/kg of DM)*	142	187	151

*calculated values

### Sample collection

Milking of lactating cows occured twice daily (0500 and 1500 h) in a 2 × 6 herringbone milking parlor (DeLaval). Milk yields of each cow were recorded automatically and stored in data files. Representative milk samples (50 mL) from every individual cow comprised two consecutive milking procedures (one evening and next morning milking) and were collected twice weekly. Milk sampling at 1, 5 and 14 wk postpartum occurred at days 5.1 ± 1.6, 29.7 ± 1.9, and 92.7 ± 1.9 (means ± SE), respectively. At 3 wk prepartum (21.1 ± 6.0 d prepartum) and 1, 5 and 14 wk postpartum (3.7 ± 1.5, 30.9 ± 1.9 and 94.2 ± 2.6 d postpartum), blood samples of the dammed vena jugularis were drawn using sterile 20 G canulas and lithium heparin tubes (Greiner bio-one, Kremsmunster, Austria). Blood sampling happened before morning feeding between 0730 and 0900 h. Tubes were kept on ice until subsequent centrifugation (2000 × *g*; 15 min). Then, plasma was transferred into 1.5 mL tubes (Greiner bio-one, Frickenhausen, Germany) and stored in aliquots at -20°C until analysis. Furthermore, liver biopsies were taken at 3 wk prepartum (20.4 ± 5.8 d prepartum), and 1, 5 and 14 wk postpartum (3.8 ± 1.4, 31.5 ± 2.1 and 94.9 ± 2.9 d postpartum). For this purpose, cows were separated and fixed after morning milking before feeding between 0700 and 0900 h. The liver biopsy site on the right side of the cow between the 11th and 12th ribs on a line between the olecranon and the tuber coxae was shaved and disinfected before a local subcutaneous anesthesia with 5 mL Isocaine 2% (Procainhydrochloride/Epinephrin, Selectavet, Weyarn/Holzolling, Germany) was performed. Then an autoclaved canula was introduced as a duct for the sterile 14 G biopsy needle (Dispomed Witt oHG, Gelnhausen, Germany) and about 50 mg of liver tissue were removed and immediately snap-frozen in liquid nitrogen. Samples were stored at -80°C until further analysis. The biopsy site was treated with wound spray and animals were kept separated for one day.

### Sample analysis

Milk protein and milk fat contents were analyzed by infrared spectrophotometry (MilkoScan-FT-6000, Foss Analytical A/S, Hillerod, Denmark) at the laboratory of Milchprüfring Bayern e.V., Wolnzach, Germany. NEFA and BHBA were determined in the thawed plasma samples using commercial available kits [NEFA-HR(2) and Autokit 3-HB, obtained from Wako Chemicals GmbH, Neuss, Germany]. Lipids from liver biopsy samples were extracted with a mixture of n-hexane and isopropanol (3:2, vol/vol) [22]. An aliquot of the extracts containing 25-50 nmoles of TAG was pipetted into a glass vial (1.5 ml), and the solvent was evaporated by vacuum. The lipids were resolved in a 20 μl portion of a 1:1-mixture of chloroform and Triton X-100 [23], and again the solvent was evaporated. Then 1 ml of commercially available enzymatic TAG kit reagent (Fluitest TG, Analyticon Biotechnologies AG, Lichtenfels, Germany) was added, and after incubation-according to the instruction of the manufacturer-the TAG content was determined by colorimetry.

### Energy balance

For calculation of the average daily energy balance of every individual cow, energy intake was calculated from the mean daily intake of PMR, SUPP and CONC and the corresponding energy contents (MJ NEL). Body weights (BW) of the cows were automatically recorded daily by electronic scales installed in the feeding stations. Using the weekly mean BW of the cows, energy requirements for maintenance were calculated according to the German Society of Nutrition Physiology [21]. Those for milk production were calculated on the basis of weekly means of daily milk yield, milk protein content and milk fat content [21]. Changes in body composition were not considered in energy balance evaluation.

### Carnitine analysis

Concentrations of total carnitine, free carnitine, acetylcarnitine and propionylcarnitine in plasma, milk and liver tissue were determined by tandem mass spectrometry [24,25]. In brief, freeze dried tissue samples were extracted with methanol:water (2:1 v/v) by homogenization (Tissue Lyser, Qiagen, Hilden, Germany), followed by sonification for 20 min and incubation at 50°C for 30 min in a shaker. After centrifugation (13000 × *g*, 10 min) 20 μL of the supernatant were added with 100 μL methanol containing the internal standards, mixed, incubated for 10 min, and centrifuged (13000 × *g*, 10 min). Plasma and milk samples were handled at 4°C in the same manner as the supernatant after tissue extraction. The final supernatants were used for quantification of the compounds by a 1100 series HPLC (Agilent Technologies, Waldbronn, Germany) equipped with a Kromasil 100 column (125 mm × 2 mm, 5 μm particle size, CS-Chromatographie Service Langerwehe, Germany) and an API 2000 LC-MS/MS-System (Applied Biosystems, Darmstadt, Germany). As eluents, methanol and a methanol:water:ACN:acetic acid mixture (100:90:9:1 v/v/v/v) were used.

### RNA isolation and quantitative real-time PCR (qPCR)

Total RNA was isolated from liver biopsies using Trizol reagent (Invitrogen, Karlsruhe, Germany) according to the manufacturer's protocol. RNA from 10 mg of each sample was isolated within one week after finishing the trial. Isolated RNA was preserved at -80°C until use. To estimate RNA concentration and purity, the optical density at 260 and 280 nm, respectively, was determined using an Infinite 200 M microplate reader and a NanoQuant Plate (both from Tecan, Mannedorf, Switzerland). The A260/A280 ratios were 1.96 ± 0.05. In addition, the optical density at 230 nm was determined and the A260/A230 ratios were calculated to control for the presence of contaminations such as guanidine thiocyanate. Although the A260/A230 ratio of some samples was below 2.0 indicating the presence of guanidine thiocyanate, it has been shown that guanidine thiocyanate has no measurable effect on downstream applications such as RT-qPCR until concentrations of more than 100 mM [26] Moreover, RNA quality was assessed by 1% agarose gel electrophoresis. RNA was judged as suitable for only if the samples exhibited intact bands corresponding to the 18S and 28S ribosomal RNA subunits. cDNA was synthesized after RNA extraction from 1.2 μg of total RNA using 100 pmol dT18 primer (Eurofins MWG Operon, Ebersberg, Germany), 1.25 μL 10 mmol/L dNTP mix (GeneCraft, Ludinghausen, Germany), 5 μL buffer (Fermentas, St. Leon-Rot, Deutschland), and 60 units M-MuLV Reverse Transcriptase (MBI Fermentas, St. Leon-Rot, Germany) at 42°C for 60 min, and a final inactivating step at 70°C for 10 min in Biometra Thermal Cycler (Whatman BiometraR, Göttingen, Germany). Subsequently, cDNA was stored in aliquots at -20°C. For the standard curve a cDNA pool of all samples was made. qPCR was performed using 2 μL cDNA combined with 18 μL of a mixture composed of 10 μL KAPA SYBR FAST qPCR Universal Mastermix (Peqlab, Erlangen, Germany), 0.4 μL each of 10 μM forward and reverse primers and 7.2 μL DNase/RNase free water in 0.1 mL tubes (Ltf Labortechnik, Wasserburg, Germany). Gene-specific primer pairs obtained from Eurofins MWG Operon (Ebersberg, Germany) were designed using Primer3 and BLAST. Features of primer pairs are listed in Table 2. All primer pairs were designed to have annealing temperatures of about 60°C, and, if possible, both primers of a primer pair were designed to be located in different exons. qPCR runs were performed with a Rotorgene 2000 system (Corbett Research, Mortlake, Australia), and included all samples and a 5 point relative standard curve plus the non-template control (NTC). The qPCR protocol was as follows: 3 min at 95°C, followed by 40 cycles of a two-step PCR consisting of 5 sec at 95°C (denaturation) and 20 sec at 60°C (annealing and extension). Subsequently, melting curve analysis was performed from 50°C to 95°C to verify the presence of a single PCR product. In addition, the amplification of a single product of the expected size was confirmed using 2% agarose gel electrophoresis stained with GelRedTM nucleic acid gel stain (Biotium, California, USA). Data on qPCR performance for each genes measured are also shown in Table 2. Reference gene stability was determined by performing GeNorm analysis [27] which is based on calculation of a reference gene-stability measure *M*. Out of six tested potential reference genes, the three reference genes with the lowest *M *values have the most stable expression and are used to calculate the GeNorm normalization factor. Therefore, all Ct values were transformed into relative quantification data by using the 2^-ΔΔCt ^equation [28]. Using the GeNorm normalization factor, relative expression levels were calculated, and from normalized expression data, means and SE were computed for samples of the same lactation week. The mean of 3 wk prepartum was set to 1 and relative expression ratios of 1, 5 and 14 wk postpartum are expressed as fold changes compared to 3 wk prepartum.

**Table 2:** Characteristics and performance data of the primers used for reference gene-stability measure *M *and quantitative real-time PCR analysis

Gene	Forward primer (from 5' to 3')	PCR product	NCBI GenBank	Slope	R^2^	Efficiency	*M*
	Reverse primer (from 5' to 3')						
*Reference genes*							
ACTB	ACTTGCGCAGAAAACGAGAT	120	AY141970	-0.30	0.99	1.99	0.039
	CACCTTCACCGTTCCAGTTT						
SDHA	GCAGAACCTGATGCTTTGTG	185	NM_174178	-0.24	0.99	1.74	0.048
	CGTAGGAGAGCGTGTGCTT						
ATP5B	GGACTCAGCCCTTCAGCGCC	229	NM_175796.2	-0.16	0.99	1.44	0.039
	GCCTGGTCTCCCTGCCTTGC						
RPS9	GTGAGGTCTGGAGGGTCAAA	108	BC148016	-0.31	0.99	2.04	0.040
	GGGCATTACCTTCGAACAGA						
PPIA	GGCAAATGCTGGCCCCAACACA	87	NM_178320.2	-0.34	0.99	2.13	0.034
	AGTACCACGTGCTTGCCATCCA						
RPL12	CACCAGCCGCCTCCACCATG	84	NM_205797.1	-0.35	0.99	2.25	0.036
	CGACTTCCCCACCGGTGCAC						
*Target genes*							
ACADM	GCGAGTACCCTGTCCCATTA	243	NM_001075235	-0.29	0.99	1.93	
	CCTCAGTCATTCTCCCCAAA						
ACOX1	CCATTGCCGTCCGATACAGT	99	BC102761	-0.27	0.96	1.88	
	GTTTATATTGCTGGGTTTGATAATCCA						
ALDH9A1	CAGGATTCGGCAGAGAGAAC	229	NM_001046423	-0.28	0.99	1.90	
	TGAGCCATGAAGAGCATCAC						
BBOX1	TCCAGCTGCCTACTCTGGAT	292	BC149884.1	-0.28	0.99	1.91	
	AGCTGAACCTTACCCCAGGT						
CD36	GCATTCTGAAAGTGCGTTGA	179	BC103112	-0.28	0.98	1.91	
	CGGGTCTGATGAAAGTGGTT						
CPT1A	CAAAACCATGTTGTACAGCTTCCA	111	FJ415874	-0.32	0.99	2.09	
	GCTTCCTTCATCAGAGGCTTCA						
HMGCS2	GCCCAATATGTGGACCAAAC	209	NM_001045883	-0.29	0.99	1.96	
	ATGGTCTCAGTGCCCACTTC						
PPARA	GGTGGAGAGTTTGGCAGAACCAGA	168	BT020756.1	-0.23	0.99	1.70	
	TCCCACTGCCCAGCTCCGATC						
SLC22A5	CACAGTGGTCAGGAACATGG	181	BC105377	-0.28	0.99	1.89	
	AATGGTGTCTGGGAGTGGAG						
SLC27A1	CTGAAGGAGACCTCCACAGC	208	NM_001033625.2	-0.30	0.99	1.99	
	GTGGTACAGGGGCAGACAGT						
TMLHE	TGGCAGGACACTGCTAGTTG	222	NM_001076064.1	-0.31	0.99	2.05	
	GACAGCCCGGTCATAGTTGT						

### Statistics

Data were statistically evaluated by a generalized linear model, including the factors time point of sampling, animal, parity number and the interactions between these factors, using the Minitab Statistical Software Release 13.0 (Minitab, State College, PA, USA). Prior to statistical analysis, all data were checked for normality and outliers before statistical analysis. As there was no significant effect of animal and parity number on the parameters investigated, only the effects of time point of sampling are reported in the results section. Linear regression models for relationships of carnitine concentrations in liver tissue with metabolic parameters at the different time points were subjected to analysis by fitted line plots. The significances of differences between the groups over time were analyzed by Fisher's multiple range test. Differences were regarded as significant for *P *< 0.05.

## Results

### Dry matter intake, performance and energy balance in dairy cows in the transition period and at different stages of lactation

Dry matter intake of the cows 1 wk postpartum was similar to that 3 wk prepartum and increased thereafter towards 5 and 14 wk postpartum (Table 3). The onset of lactation led to a strong negative energy balance (Table 3). Energy balance that was strongest negative in 1 wk postpartum was then improving. At 14 wk postpartum, the cows returned to a slight positive energy balance (Table 3). Milk yield was increasing from 1 to 5 wk postpartum and was thereafter declining towards 14 wk postpartum (Table 3). Milk fat and milk protein contents were highest at 1 wk postpartum and were thereafter declining (Table 3).

**Table 3:** Performance of dairy cows in the transition period and at different stages of lactation

Variable	3 wk prepartum	1 wk postpartum	5 wk postpartum	14 wk postpartum	wks 1 to 14 postpartum	*P*-value
Dry matter intake (kg/d)	13.4^c ^± 0.33	13.4^c ^± 0.48	18.4^b ^± 0.62	20.4^a ^± 0.53	18.5 ± 0.49	< 0.001

Milk yield (kg/d)	-	28.6^c ^± 0.76	37.5^a ^± 0.90	32.0^b ^± 0.81	32.7 ± 0.67	< 0.001

FCM* (kg/d)	-	39.2^a ^± 1.39	40.9^a ^± 1.51	33.0^b ^± 1.03	37.7 ± 0.87	< 0.001

Milk fat (%)	-	6.40^a ^± 0.25	4.51^b ^± 0.20	4.11^b ^± 0.13	5.00 ± 0.17	< 0.001

Milk protein (%)	-	4.09^a ^± 0.09	2.85^c ^± 0.05	3.13^b ^± 0.06	3.35 ± 0.08	< 0.001

Energy balance^† ^(MJ NEL/d)	46.9^a ^± 2.42	-64.9^d ^± 5.15	-30.4^c ^± 3.05	3.89^b ^± 2.11	-12.5 ± 5.52	< 0.001

Energy intake^† ^(% of requirement)	215.1 ± 7.92	58.9 ± 2.45	82.3 ± 2.22	102.1 ± 2.10	81.1 ± 2.63	< 0.001

Values are mean ± SE (n = 20)

^a, b, c, d ^Means with different superscripts differ significantly (*P *< 0.05)

* Corrected for 4% milk fat content

^† ^Calculated value

### Relative mRNA abundances of PPARA and genes involved in fatty acid uptake, fatty acid oxidation and ketogenesis in the liver of dairy cows in the transition period and at different stages of lactation

**Figure 1:**
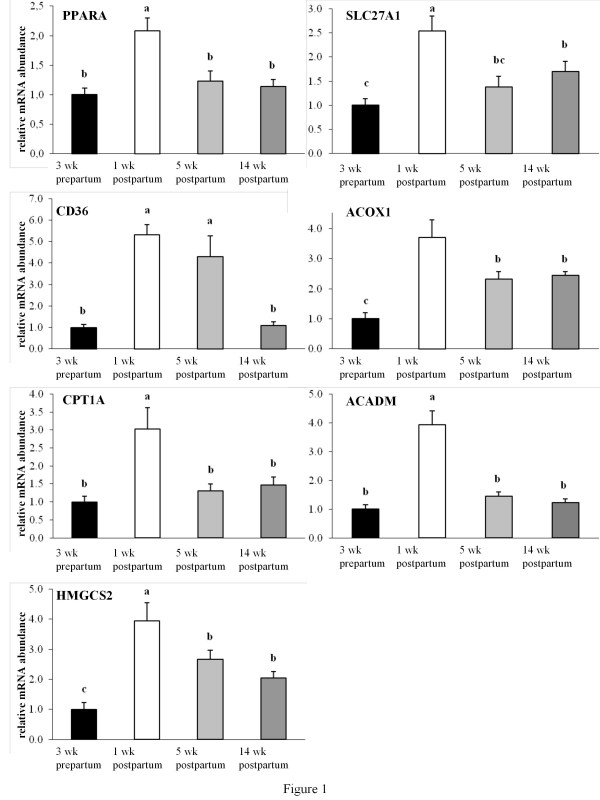
**Relative mRNA abundances of PPARA and genes involved in fatty acid uptake, fatty acid oxidation and ketogenesis**. Relative mRNA abundances of PPARA and genes involved in fatty acid uptake (SLC27A1, CD36), fatty acid oxidation (ACOX1, CPT1A, ACADM) and ketogenesis (HMGCS2) in the liver of dairy cows in the transition period and at different stages of lactation; bars represent means ± SE (n = 20) and are expressed relative to the mRNA abundance at 3 wk prepartum. ^a, b, c ^Bars with different superscripts differ significantly (*P *< 0.05).

The relative mRNA abundance of PPARA in the liver was increased from 3 wk prepartum to 1 wk postpartum (*P *< 0.05) and was thereafter declining, reaching values at 5 wk and 14 wk postpartum similar with that of 3 wk prepartum (Figure 1). In accordance with the expression pattern of PPARA, target genes involved in fatty acid uptake (SLC27A1, CD36), mitochondrial and peroxisomal β-oxidation (ACOX1, CPT1A, ACADM) and ketogenesis (HMGCS2) were rising from 3 wk prepartum to 1 wk postpartum (*P *< 0.05, Figure 1). From 1 to 5 and 14 wk postpartum, mRNA abundances of these genes, with the only exception of CD36, were declining (*P *< 0.05, Figure 1). mRNA abundances of ACOX1, SLC27A1 and HMGCS2 remained at a higher level at 5 and 14 wk postpartum than at 3 wk prepartum (*P *< 0.05, Figure 1). In contrast, mRNA abundances of CPT1A and ACADM, two enzymes of mitochondrial β-oxidation, returned to levels similar to those at 3 wk prepartum (Figure 1).

### Relative mRNA abundances of genes involved in carnitine synthesis and carnitine uptake in the liver of dairy cows in the transition period and at different stages of lactation

**Figure 2:**
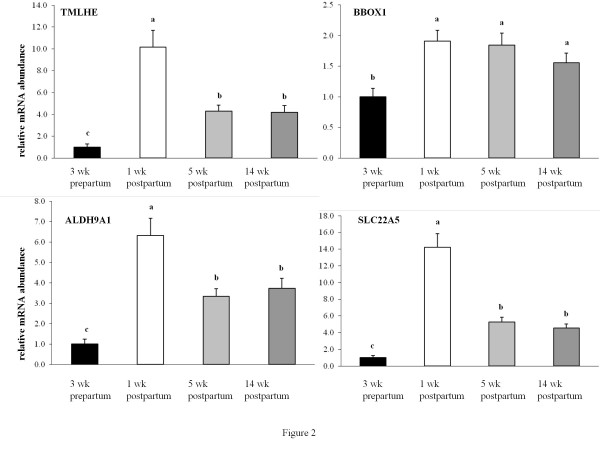
**Relative mRNA abundances of genes involved in carnitine synthesis and carnitine uptake**. Relative mRNA abundances of genes involved in carnitine synthesis (TMLHE, ALDH9A1, BBOX1) and carnitine uptake (SLC22A5) in the liver of dairy cows in the transition period and at different stages of lactation; bars represent means ± SE (n = 20) and are expressed relative to the mRNA abundance at 3 wk prepartum. ^a, b, c ^Bars with different superscripts differ significantly (*P *< 0.05).

mRNA abundances of the two genes involved in the formation of γ-butyrobetaine-the precursor of carnitine-TMLHE and ALDH9A1, were strongly (10-, and 6-fold, resp.) increased from 3 wk prepartum to 1 wk postpartum (*P *< 0.05, Figure 2). mRNA abundance of BBOX1, the enzyme which converts γ-butyrobetaine into carnitine, was moderately (1.8-fold) increased from 3 wk prepartum to 1 wk postpartum (*P *< 0.05, Figure 2). mRNA abundance of SLC22A5, the most important carnitine transporter, was strongly (13-fold) increased from 3 wk prepartum to 1 wk postpartum (*P *< 0.05, Figure 2). With the exception of BBOX1, mRNA abundances of all these genes involved in carnitine synthesis pathway and carnitine uptake were declining from 1 wk to 5 and 14 wk postpartum. Nevertheless, mRNA abundances of these genes in 5 and 14 wk postpartum remained at levels higher than those at 3 wk prepartum (*P *< 0.05, Figure 2).

### Concentrations of carnitine in liver, plasma and milk of dairy cows in the transition period and at different stages of lactation

In the liver, free carnitine was nearly the exclusive form of carnitine whereas concentrations of carnitine esters (< 1 nmol/g) were only slightly above the detection limit and are therefore not reported. Liver free carnitine concentration was rising from 3 wk prepartum to 1 wk postpartum (*P *< 0.05) and was thereafter falling to levels below those observed at 3 wk prepartum (Table 4). In plasma, the concentration of free carnitine was strongly decreasing from 3 wk prepartum to 1 wk postpartum and was thereafter rising to values which remained however below those observed 3 wk prepartum (*P *< 0.05, Table 4). In contrast, the concentration of carnitine esters in plasma was increasing from 3 wk prepartum to 1 wk postpartum (*P *< 0.05) and was thereafter decreasing; plasma concentration of carnitine esters at 14 wk postpartum was even lower than that 3 wk prepartum (*P *< 0.05, Table 4). Plasma concentration of total carnitine decreased from 3 wk prepartum to 1 wk postpartum (*P *< 0.05) and remained thereafter at a constant level (Table 4). Concentrations of free, esterified and total carnitine in milk were highest at 1 wk postpartum; concentrations were thereafter decreasing and were similar at 5 wk and 14 wk postpartum (Table 4).

**Table 4:** Concentrations of carnitine in liver biopsy, plasma and milk samples of dairy cows in the transition period and at different stages of lactation

	3 wk prepartum	1 wk postpartum	5 wk postpartum	14 wk postpartum	*P*-value
*Liver tissue*					
free carnitine, nmol/g wet weight	37.4^b ^± 4.53	55.6^a ^± 4.09	26.0^c ^± 1.76	18.2^c ^± 1.26	< 0.001

*Plasma*					
free carnitine, μmol/L	3.82^a ^± 0.25	1.40^c ^± 0.10	2.18^b ^± 0.20	2.35^b ^± 0.17	< 0.001
carnitine esters*, μmol/L	2.07^b ^± 0.10	2.54^a ^± 0.12	2.45^ab ^± 0.18	1.64^c ^± 0.11	< 0.001
total carnitine^†^, μmol/L	5.89^a ^± 0.33	3.94^b ^± 0.17	4.64^b ^± 0.32	3.99^b ^± 0.26	< 0.001

*Milk*					
free carnitine, μmol/L	-	84.5^a ^± 5.92	50.3^b ^± 4.34	60.2^b ^± 2.65	< 0.001
carnitine esters*, μmol/L	-	131.1^a ^± 12.1	65.9^b ^± 6.23	41.3^c ^± 2.60	< 0.001
total carnitine^†^, μmol/L	-	215.5^a ^± 16.6	116.2^b ^± 7.92	101.5^b ^± 3.93	< 0.001

Values are mean ± SE (n = 20)

^a, b, c ^Means with different superscripts differ significantly (*P *< 0.05)

*Sum of acetyl- and propionyl carnitine, ^†^Sum of free carnitine and carnitine esters

### Correlations between liver carnitine concentration and phenotypic measures (plasma NEFA and BHBA, hepatic concentration of TAG)

In order to assess whether plasma NEFA concentrations influence liver carnitine concentrations, we calculated a linear regression between plasma NEFA and liver carnitine concentrations. As expected, plasma NEFA concentrations were strongly increasing from 3 wk prepartum to 1 wk postpartum and were thereafter declining (Table 5). At all the three time points considered during lactation (1 wk, 5 wk, 14 wk postpartum), a significant positive correlation between plasma NEFA concentration and liver carnitine concentration was observed (*P *< 0.05, Table 6). In contrast, an inverse correlation between plasma NEFA concentration and liver carnitine concentration was observed at 3 wk prepartum. In order to elucidate whether liver carnitine status influences hepatic TAG accumulation or ketone body formation, we calculated linear regressions between liver carnitine concentrations and liver TAG concentrations or plasma BHBA concentrations. As expected, liver TAG concentration was highest at 5 wk postpartum and BHBA concentrations were highest at 1 wk and 5 wk postpartum (Table 5). However, no significant correlations between liver carnitine concentrations and liver TAG or plasma BHBA concentrations emerged, both pre- and postpartum (Table 6).

**Table 5:** Metabolic parameters in liver biopsy and plasma samples of dairy cows in the transition period and at different stages of lactation

	3 wk prepartum	1 wk postpartum	5 wk postpartum	14 wk postpartum	*P*-value
Plasma NEFA (μmol/L)	103^d ^± 10	864^a ^± 47	276^b ^± 16	171^c ^± 11	< 0.001

Liver triglyceride (mg/g wet weight)	4.56^c ^± 0.45	23.5^b ^± 2.6	64.3^a ^± 10.3	3.18^c ^± 0.28	< 0.001

Plasma BHBA (μmol/L)	484^b ^± 23	707^a ^± 42	724^a ^± 46	521^b ^± 24	< 0.001

Values are mean ± SE (n = 20).

^a, b, c, d ^Means with different superscripts differ significantly (*P *< 0.05)

**Table 6:** Linear regression parameters for the relationship of concentration of free carnitine in liver biopsy samples (nmol/g wet weight) as predictor variable with different metabolic parameters of dairy cows in the transition period and at different stages of lactation as response variables

Response variable		3 wk prepartum	1 wk postpartum	5 wk postpartum	14 wk postpartum
Plasma NEFA (μmol/L)	Intercept	152.7	285.7	115.7	81.4
	Slope	-1.17	10.2	5.86	4.66
	R^2^	0.29	0.64	0.38	0.26
	*P*-value	0.015	< 0.001	0.008	0.035

Liver triglyceride (mg/g wet weight)	Intercept	3.07	30.5	44.1	3.81
	Slope	0.04	-0.14	0.65	-0.05
	R^2^	0.15	0.09	0.01	0.09
	*P*-value	0.112	0.291	0.673	0.305

Plasma BHBA (μmol/L)	Intercept	562.8	605.3	603.7	503.3
	Slope	-1.84	1.43	4.56	0.60
	R^2^	0.13	0.02	0.03	0.00
	*P*-value	0.113	0.621	0.505	0.907

## Discussion

This study was performed to investigate the hypothesis that the onset of lactation in dairy cows leads to an up-regulation of genes involved in hepatic carnitine synthesis and uptake of carnitine. As expected, the transition from late pregnancy to early lactation was associated with a strong negative energy balance resulting in increased concentrations of NEFA and BHBA in plasma and an increase in hepatic TAG concentration. Similar metabolic changes during the periparturient period in cows have been observed in many other studies [e.g. [16,18,29]]. In agreement with a recent study [16], we observed that the negative energy balance occurring at early lactation was associated with an increased expression of several PPARA target genes involved in fatty acid uptake, mitochondrial and peroxisomal fatty acid oxidation and ketogenesis. Although we were not able to give direct proof of PPARA activation due to small liver sample amount available, an up-regulation of various PPARA target genes is indicative of an activation of that transcription factor in the liver. While there is less research about activation of PPARA in cattle, studies in other species such as rodents or pigs have clearly shown that increased plasma NEFA concentrations, induced by energy deprivation, are leading to an activation of PPARA in liver and other tissues [12,13,30]. As long-chain fatty acids are also acting as agonists of PPARA in bovine cells [15], it seems justified to speculate that high plasma NEFA concentrations in dairy cows during early lactation were causing an activation of PPARA in the liver. It should be noted, however, that in opposite to our study and the study of Loor et al. [16], there are also studies which did not observe an up-regulation of PPARA and PPARA target genes in the liver of dairy cattle during early lactation, particularly when cows had prepartum a caloric intake in excess of 100% of their energy requirement [31,32]. The lack of up-regulation of PPARA during early lactation in these studies has been explained by a hepatic inflammatory response, induced by an excessive prepartum caloric intake, which decreased pre- and postpartum hepatic expression of expression of PPARA [32].

In accordance with the hypothesis of this study, we observed for the first time that the transition from late pregnancy to early lactation leads to an up-regulation of various genes involved in carnitine synthesis (ALDH9A1, TMLHE, BBOX1) and carnitine uptake (SLC22A5) in the liver of cows at 1 wk postpartum. As all these genes are PPARA target genes with functional PPREs identified in their promoters or first introns [32-35], we assume that the up-regulation of these genes in the liver of dairy cows at 1 wk postpartum was caused by a potential activation of PPARA. The finding of positive correlations between plasma concentrations of NEFA, which might be regarded as natural agonists, and hepatic carnitine concentrations during lactation supports a role of PPARA in the regulation of genes of carnitine synthesis and uptake.

The present study confirms previous studies in showing that liver carnitine concentration is increasing during the transition from late pregnancy to lactation and is thereafter continuously decreasing to values similar or even below those observed in pregnancy [18,19]. In the study of Carlson et al. [19], hepatic total carnitine concentrations were around 1.6-fold higher on 2 d of lactation compared to 3 wk prepartum, while values at d 28 were even lower than those 3 wk prepartum. In the study of Grum et al. [18], concentrations of acid-soluble carnitine (free carnitine plus short-chain acyl carnitine) sharply increased from 3 wk prepartum to 1 d postpartum and returned to prepartum values at d 21 postpartum. As carnitine is synthesized from trimethyllysine released primarily from turnover of skeletal muscle proteins, Grum et al. [18] suggested that an increased hepatic carnitine concentration at 1 d postpartum might be due to an enhanced catabolism of muscle protein in this early stage of lactation. Although we have not directly measured carnitine synthesis and uptake into cells, the findings of increased mRNA expression of genes of carnitine synthesis and uptake suggest that increased hepatic carnitine concentrations at early lactation could also be due, at least in part, to an increased carnitine synthesis in the liver and an increased uptake of carnitine from blood into the liver. This suggestion is supported by studies in rodents and pigs which found that an up-regulation of enzymes of carnitine synthesis in the liver, caused either by treatment with PPARA agonists or by energy deprivation, leads to an increased hepatic carnitine concentration without changing the concentrations of BB or TML, the precursors of carnitine synthesis [7,8,36,37]. The finding that the concentration of free carnitine in plasma is strongly decreasing from 3 wk prepartum to 1 wk postpartum fits into this suggestion. Studies in rodents have shown that an up-regulation of SLC22A5 by treatment with PPARA agonists or by energy deprivation leads to a reduction of plasma carnitine concentration, due to an increased transport of carnitine from plasma into tissues [6,8,36]. The decrease in plasma carnitine concentrations during early lactation might be in part explained by the transfer of carnitine from plasma into the milk in mammary gland. Nevertheless, with respect to the findings in rodents, we assume that reduced plasma carnitine concentrations in early lactation could also be caused by an increased uptake of free carnitine from plasma into tissues, including the liver. In accordance with present study, Carlson et al. [19] also observed a strong reduction of plasma carnitine concentration from 21 d prepartum to 9 or 27 d postpartum. Those authors [19] also found that carnitine concentration in skeletal muscle is not changing significantly during transition from pregnancy into lactation. This finding also agrees with studies in rodents which show that energy deprivation does not influence muscle carnitine concentrations [7,36].

Furthermore, we observed that milk carnitine concentration is highest at 1 wk postpartum and is thereafter decreasing to 5 wk and 14 wk postpartum. This finding agrees with the study of Carlson et al. [19] which found a strong reduction of milk carnitine concentration from 2 wk to 6 wk postpartum. It is possible that the high carnitine concentration in milk at wk 1 postpartum is due to the strong negative energy balance of the cows. Carlson et al. [38] found that energy restriction of cows increased milk carnitine concentrations. In the mammary gland, carnitine is secreted into the milk by several transporters (SLC22A4, SLC22A5, OCTN3, SLC6A14, SLC6A10) [39]. Possibly, one or more of these transporters are up-regulated in a state of a negative energy balance. Studies in rats also found a reduction of milk carnitine concentration from early to later stage of lactation, due to a down-regulation of SLC22A5, OCTN3 and SLC6A14 [39].

Ketosis and fatty liver are two diseases in dairy cows during early lactation which are linked to hepatic fatty acid oxidation [40]. As carnitine is involved in β-oxidation due to its role in the transport of long chain fatty acids into the mitochondrion, it was interesting to explore whether plasma ketone body concentrations or hepatic TAG concentrations are correlated with hepatic carnitine concentrations. The observation that there were no correlations between hepatic carnitine concentration and both, plasma BHBA and hepatic TAG concentrations, at 1, 5 and 14 wk postpartum suggests that the availability of carnitine in the liver had no influence on ketogenesis and TAG accumulation in the liver. The observation that hepatic carnitine concentration does not correlate with BHBA concentration is in accordance with a study showing that carnitine supplementation does not influence plasma BHBA concentration in early lactating dairy cattle [41]. The finding that the activity of CPT1A does not play a primary role in the etiology of ketosis [42] is another indication that the availability of carnitine, which acts as a cofactor of that enzyme, is not a key factor in the production of ketone bodies. Accumulation of TAG in the liver is explained by the observation that the capacity of bovine liver tissue to convert fatty acids to esterified products is strongly increased during the early postnatal period, whereas fatty acid oxidation is only slightly increased, meaning that NEFA from mobilization are directed towards conversion to TAG [43]. The finding that there was no correlation between hepatic carnitine concentration and hepatic TAG concentration thus indicates that a higher hepatic carnitine concentration did not stimulate hepatic fatty acid oxidation. This indication is, however, in contradiction to some in vitro and in vivo studies in dairy cows. In vitro studies using bovine liver slices have shown that addition of carnitine enhances the oxidation of palmitate [44,45]. Moreover, postruminal infusion of carnitine enhanced palmitate oxidation and decreased liver lipid accumulation in cows with experimentally induced negative energy balance [41]. These studies suggested that carnitine might be the rate-limiting factor of hepatic β-oxidation in dairy cows during the periparturient period and that carnitine supplementation might prevent the development of a fatty liver.

## Conclusions

The present study shows for the first time that hepatic mRNA abundances of genes involved in carnitine synthesis and cellular uptake of carnitine in dairy cows are increased during the transition from late pregnancy to lactation. An up-regulation of genes involved in carnitine biosynthesis and uptake could contribute to elevated hepatic carnitine concentration in early lactation observed in this and previous studies.

## Authors' contributions

GS: conducted the animal experiment, performed the statistical analyses and wrote the manuscript. JK: performed the PCR analyses. FH and SG: performed the carnitine analyses and helped to draft the manuscript; FJS: participated in the design of the study and supervised the animal experiment; RR: supervised PCR analyses. GIS: supervised the carnitine analyses. KE: conceived of the study, and participated in its design and coordination and helped to draft the manuscript. All authors read and approved the final manuscript.
